# The impact of clinical education on knowledge and attitudes towards brain death among Polish medical students – a cross-sectional study

**DOI:** 10.1186/s12909-023-04637-y

**Published:** 2023-09-14

**Authors:** Krzysztof Kowalski, Julia Marschollek, Marta Nowakowska-Kotas, Sławomir Budrewicz

**Affiliations:** 1 Department of Psychiatry, Wroclaw Medical University, wyb. L. Pasteura 10, Wroclaw, 50-367 Poland; 2 Department of Neurology, Wroclaw Medical University, Borowska 213, Wroclaw, 50-556 Poland

**Keywords:** Medical students, Brain death, Knowledge

## Abstract

**Background:**

Understanding brain death is essential for progress in organ transplantation; however, it remains a challenging ethical matter. In 2019, Poland revised its legislation on brain death to align with international standards. This study aimed to evaluate the knowledge and worldview concerning brain death among Polish medical students, categorised according to their stage of education.

**Methods:**

An online questionnaire was administered to 169 medical students from four Polish medical universities. The participants were divided into preclinical (*n* = 94) and clinical (*n* = 75) groups. The questionnaire consisted of two parts, with the first part comprising 13 questions focusing on knowledge about brain death and the process of its determination. The second part contained six questions related to the participants' worldview regarding brain death, particularly concerning organ transplantation.

**Results:**

The average score obtained by the respondents was 7.53 (± 2.35; min. 1, max. 13) in knowledge checking part of the developed questionnaire (maximal score:13). Students in the clinical stage of their education achieved significantly higher scores compared to preclinical students (mean 8.84; ± 1.89 vs mean 6.49; ± 2.15; *p* < 0.001). Significant correlations were found between the results of the knowledge part of the questionnaire and responses to worldview questions.

**Conclusions:**

The stage of education influenced the knowledge of brain death among medical students, although the overall test scores were unsatisfactory. Higher test scores were associated with worldview responses indicating compliance with the current legislation in Poland and evidence-based medicine.

**Supplementary Information:**

The online version contains supplementary material available at 10.1186/s12909-023-04637-y.

## Background

Brain death, although currently strictly defined both formally and legally, arouses considerable controversy in terms of the development of medicine, especially among the general public [1]. The correct diagnosis of brain death is essential for transplantology, as it allows for the cessation of ineffective treatment procedures and the collection of organs for potential transplantation. Knowledge of brain death and attitudes towards organ donation among medical staff and relatives of patients influence the donation process [2]. The awareness of such state as brain death and its importance for organ donation is low to moderate in citizens [3, 4], and knowledge of young pre- specialist doctors, senior doctors of various specialities as well as some anesthesiologists, which the exception of those working in intensive care units, about the up-to-date definitions of brain death, is unsatisfactory [5–8]. In addition, attitudes towards the definition of brain death and its impact on organ donation appears exhibit notable discrepancies, extending beyond just professional roles [8–10].

The definition of brain death has developed over the decades. This term was first introduced in 1968 in the report "A Definition of Irreversible Coma". The Committee of the Harvard Medical School to Examine the Definition of Brain Death clarified the definition of brain death as being 'the irreversible, permanent cessation of whole-brain activity as determined by comprehensive studies [11]. In 1981, the President's Commission for the Study of Ethical Problems in Medicine and Biomedical and Behavioral Research developed The Uniform Determination of Death Act (UDDA), which recognised death as being the irreversible interruption of circulation and respiration or irreversible cessation of all brain functions, especially the death of the brain stem. Moreover, this loss of function cannot return spontaneously or through medical intervention [12]. At the same time, the death of the brain stem does not necessarily mean the immediate death of all brain cells. Recently, a new, modified definition of brain death has been established by an international panel of experts, the World Brain Death Project, which has been widely accepted worldwide. The brain death adjudication process consists of two stages – clinical examination and ancillary diagnostic tools – the results of which, according to the latest guidelines, should leave no room for error [13]. According to world data, ancillary diagnostic tools have 100% sensitivity and specificity for diagnosing brain death [14].

In Poland, legal regulations appeared in 1984 in the Communication of the Ministry of Health and Social Welfare, and these were then modified in amendments appearing in 1994 and 1996. Later, the Proclamation of the Minister of Health on 17 July 2007, "on the criteria and method for determining permanent, irreversible cessation of brain activity", was in force for several years. This was based on the assumption that "Death is a dissociated phenomenon (…), affecting tissues and systems at different times (…); therefore, some functions of the system or their parts may remain for some time in isolation from others which have already died." The current criteria for the diagnosis of brain death, adapted to world standards, were outlined in the Proclamation of the Minister of Health on 4 December 2019.

In recent years, many efforts have been made to determine the reasons for the low rate of transplantation from deceased donors despite the precise establishment of brain death criteria. Potential reasons for this include the religious beliefs of the deceased's relatives, the chaplains' involvement, and the medical staff's knowledge and attitude towards brain death [2, 15–17]. In turn, the process of shaping physicians' attitudes towards death is protracted and begins during their medical studies, potentially as early as in the first preclinical years [17].

The issue of brain death is included in the medical curriculum, but the effectiveness of obtaining this knowledge is subject to certain limitations [18, 19]. This study aimed to assess the knowledge and understanding/appreciation of brain death as well as their attitudes and beliefs toward brain death among Polish medical students divided by the stages of their education.

## Methods

The study was conducted in 2020 using a proprietary questionnaire. It was an online quantitative computer-assisted web interview (CAWI) survey in the form of a questionnaire to be completed on a computer by the respondent; it was voluntary and anonymous. The survey was sent to the medical student of four Polish universities: Wroclaw, Poznan, Katowice, and Warsaw. Before participating in the study, the respondents were informed about the study's objectives. During the course, the respondents first gave informed consent with the additional information that they had the opportunity to withdraw from participation at any time point without giving any reason.

The questionnaire developed by the authors consisted of two parts. The first part assessed knowledge through a test containing 13 single-choice closed questions related to understanding brain death and its assessment. Questions 1 to 9 focused on clinical aspects of brain death and its determination, while questions 10 to 13 addressed formal and legal aspects. The total possible score was 13 points. The second part of the questionnaire explored respondents' attitudes about brain death and its determination. The second part consisted of questions about the respondents' beliefs about brain death (one question) and attitudes (five questions) toward organ transplantation and its adjudication. There were five options to choose from as an answer: "I strongly disagree", "I tend to disagree", "I have no opinion", "I tend to agree", and "I strongly agree". These were assigned ordinal numbers from 1 to 5 for the correlation assessment. To present the data in the table, the responses "I strongly disagree" and "I tend to disagree" were jointly considered "Disagree with the statement", and "I strongly agree" and "I tend to agree" were considered "Agree with the statement". The entire content of the questionnaire is available as Supplementary Material 1.

Statistical analysis was performed using the STATISTICA program (StatSoft, version 13.3). The Kolmogorov–Smirnov test was applied and indicated that the data were not normally distributed. Hence, between-group differences were evaluated using the Mann–Whitney U-test. Rho–Spearman rank correlation was used to analyse correlations between continuous variables. Qualitative variables were expressed as percentages, quantitative as the means, and standard deviations (SD). The *chi*-square test determined the relationships between the compared qualitative variables. In all tests, the probability level of *p* < 0.05 was considered significant.

### Material

One hundred sixty-nine medical students from four Polish medical universities participated in the study. They were divided into two groups on the basis of the study stage. Students from years 1–3 were termed "preclinical", and students from years 4–6 were termed "clinical" because the subjects "Anesthesiology and Intensive Therapy" as well as "Neurology" and "Neurosurgery", which are essential for the research topic, are taught during the 4th, 5th or 6th years of studies depending on the medical university. The numbers for these groups are presented in Table 1.

**Table 1:** The sizes of the studied groups

Study stage	Number of respondents
Preclinical	*n* = 94 (55.6%)
Clinical	*n* = 75 (44.4%)

### Ethics

An experimental protocol, including the questionnaire, was approved by the Wroclaw Medical University Commission of Bioethics (no 733/2022, Wroclaw Medical University). All subjects provided informed consent prior to to their inclusion in the study. All methods were carried out in accordance with relevant guidelines and regulations.

## Results

### Test of knowledge

The average result obtained by the respondents was 7.53 (± 2.35; 57.9%; min. 1 max. 13). The results for individual subgroups of questions, particular questions and learning stages are presented in Tables 2 and 3.

**Table 2:** The results of the knowledge test, taking into account the subgroups of questions and study stages

	Preclinical stage N(%)	Clinical stage N%	*p*
Clinical issues	5 (55.6)	6.72 (74.6)	< 0.001
Legal issues	1.49 (37.3)	2.12 (53)	< 0.001
Combined	6.49 (49.9)	8.84 (68)	< 0.001

**Table 3:** Detailed comparison of the correctness of answers to particular questions

Issue related to brain death or its adjudication raised in the question	Correct answers		*P*
	Preclinical stage N (%)	Clinical stage N (%)	
1. Diagnostic criteria	73 (77.7)	68 (90.7)	**0.021**
2. Exclusions	60 (63.8)	54 (72)	0.258
3. Maintaining pregnancy in a mother who has been diagnosed as brain dead	81 (86.2)	70 (93.3)	0.126
4. Vegetative state and locked-in syndrome	49 (52.1)	57 (76)	**0.001**
5. Tissue death	80 (85.1)	65 (86.7)	0.772
6. Conditions for the initiation of the adjudication procedure	47 (50)	41 (54.7)	0.546
7. States excluding initiation of adjudication	29 (30.9)	54 (72)	**< 0.001**
8. Instrumental tests	15 (15.9)	35 (46.7)	**< 0.001**
9. Trunk reflexes	36 (38.3)	60 (80)	**< 0.001**
10. Adjudicating panel of brain death tests	13 (13.8)	42 (56)	**< 0.001**
11. Consent to organ donation	78 (83)	68 (90.7)	0.141
12. Possibility of donating organs in situations other than brain death	18 (19.2)	22 (29.3)	0.121
13. Formal and legal dimensions of adjudication criteria	31 (33)	27 (36)	0.681

Significant differences (*p* < 0.005) are given in bold

### Beliefs and attitudes

A detailed distribution of answers to questions related to the attitudes towards brain death is presented in Table 4.

**Table 4:** Distribution of answers to questions related to attitude towards brain death

Statement	Disagreement with the statement		Agreement with the statement		No opinion		*p*
	Preclinical stage N(%)	Clinical stage N(%)	Preclinical stage N(%)	Clinical stage N(%)	Preclinical stage N(%)	Clinical stage N(%)	
1. Brain death is an irreversible condition	1 (1.1)	0	91 (96.8)	75 (100)	2 (2.1)	0	0.168
2. Once a patient is diagnosed as brain dead, they should be promptly taken off life support (unless specific criteria are met, e.g. organ donation)	27 (28.7)	26 (34.7)	55 (58.5)	42 (56)	12 (12.8)	7 (9.3)	0.619
3. A pregnant patient who shows signs of brain death should be kept on life support if the fetus shows signs of possibility to be born	7 (7.5)	4 (5.3)	85 (90.4)	63 (84)	2 (2.1)	8 (10.7)	0.054*
4. A family should be able to question the doctor's diagnosis of brain death	62 (66)	62 (82.7)	16 (17)	9 (12)	16 (17)	4 (5.3)	**0.028**
5. Brain death is equivalent to cardiopulmonary death in the context of the death of a patient	39 (41.5)	26 (34.7)	35 (37.2)	38 (50.7)	20 (21.3)	11 (14.6)	0.196
6. Do you believe that every case of brain death should be followed by ancillary diagnostic tools (EEG, CSF flow, Electrical signals, etc.)?	10 (10.6)	20 (26.7)	69 (73.4)	38 (50.7)	15 (16)	17 (22.6)	**0.005**

Significant differences (*p* < 0.005) are given in bold

^*^ denotes almost significant differences

### The relationship between knowledge and beliefs and attitudes

A weak correlation was shown between a high total test result and agreement with statements 2 (*r* = 0.22; *p* = 0.003) and 5 (*r* = 0.16; *p* = 0.04). Moreover, a weak correlation was observed between a high test score and disagreement with statement 6 (*r* = -0.26; *p* < 0.001). The relationships between the total score and other questions about beliefs turned out to be statistically insignificant.

## Discussion

Based on our findings, there is a noteworthy enhancement in understanding the determination of brain death as medical studies progress. This improvement underscores the impact of students' engagement in clinical experiences, particularly in mastering the assessment of reflexes, and exploring topics linked to awareness and consciousness. However, our study also revealed substantial gaps in knowledge concerning diagnostic tests, procedural initiation criteria, and accurately identifying conditions suitable for organ donation. Notably, fewer than 30% of students in their advanced clinical years provided appropriate responses to these aspects. Our results are consistent with observations from Cape Town, where it was shown that students' knowledge of the issues related to brain death increased in line with the year of study while still achieving a low result in the final year of study. However, more of those students provided the appropriate medical definition of brain death than our group (96% vs 56%) [18]. In Mexican research, although more than half of medical students stated that they acquired knowledge about brain death in the second year of studies, their level of understanding of the issue of brain death was low (39% gave correct answers to the 5 essential questions in the proprietary questionnaire) [20]. In contrast, in Canadian research, knowledge about organ donation conditions did not increase significantly during the course of studies [21]. This indicates that an average graduate of medical studies, regardless of the education system, usually has incomplete knowledge about an issue as vital as brain death.

Nevertheless, changes in students' attitudes regarding brain death had a clear trend towards statements in line with legislation and evidence-based medical knowledge, along with the level of advancement of education, which could positively influence organ donation. In our group, this is especially true for claims that the family cannot question a doctor's decision (under Polish law) and that not all claims of brain-stem death require ancillary tests.

According to various studies, the acceptance of the concept of brain death and its implications for organ donation seems to vary significantly not only by professional role and country but also among students in different countries. Iranian researchers found that 93.1% of students agreed with the idea of organ donation after brain death was pronounced, thus considering brain death an irreversible condition [9]. Polish students recognised brain death as an irreversible condition to an even greater extent, while in a similar analysis among Saudi Arabian students, as many as 40% believed that brain death is a condition from which it is possible to recover [22]. Interestingly, among Canadian respondents, 76% acknowledged that someone could be neurologically deceased while their heart is still beating [23]. This understanding reflects a nuanced view of death, recognising that brain death can occur while other bodily functions, such as a beating heart, continue. It's crucial to note that the rates of acceptance appear to correlate with attitudes toward organ donation and potentially influence organ donation rates, with the highest acceptance rate in Western Europe [24].

Knowledge about brain death among medical students translates into doctors' skills in its diagnosis, which is also surprisingly low. Practising neurologists correctly answered 54% of questions on the test on neurological criteria of death [25]. The even lower level of correct answers (7.08%) regarding all necessary criteria for diagnosing brain death among various young medical trainees is concerning [5]. Certain beliefs expressed by some older specialists, including anesthesiologists, are also worrisome [6]. Considering the significant percentage of anesthesiologists admitting a lack of knowledge (50%) and expressing willingness to participate in courses on determining brain death [8], it is worth seriously considering periodic courses for individuals interested in or professionally involved in determining brain death (in Polish context: neurologists, neurosurgeons, anesthesiologists). This should ensure that the knowledge gained from studies is updated and refreshed. Among other causes, this could be one of the reasons for the low organ donation rates in Poland and worldwide. In European statistics, Poland finds itself in one of the last places in terms of organ transplants (40.6 patients per million received an organ in Poland in 2019, while in Spain, there were 114.8 such patients) [26]. The need for intervention is emphasised by the fact that at the end of December 2022, 1826 patients were on waiting lists for organ transplantation in Poland [27].

When looking for a way to increase the knowledge of medical students, it is possible to consider introducing elements of instruction about brain death as early as at the preclinical level, where, in particular, this could allow for reflection and discussion on death and dying, as well as reduce fear experienced before dissection classes [17]. A novel idea would also be the introduction of mandatory training on brain death and workshops on talking about it with a patient's family, which has already been tested on students and practising doctors, in all cases improving knowledge but also comfort when dealing with patients with suspected brain death and their relatives [25, 28, 29].

The study has some limitations, the elimination of which could result in a better interpretation. First, it does not consider the respondents' sociodemographic data or their further study plans. It would also be essential to obtain knowledge about the religion of each of the participants in the interpretation of differences in issues of belief. Another limitation is the relatively small sample size and online questionnaires, which introduces the possibility of dependent work, such as using external sources or seeking assistance from third parties while completing the test. Furthermore, online surveys are susceptible to response biases, and the sample's representativeness might be limited since it was drawn from specific universities in Poland. The cross-sectional design of the study limits the establishment of causal relationships.The choice of this study method was associated with limitations related to the epidemiology of COVID, and difficulties related to distance learning may have influenced the results obtained in the test [19]. In the future, it would be worthwhile to compare the international knowledge of medical students on brain death based on a new, standardised questionnaire, the questions of which would not include issues related to the legislation of individual countries.

## Conclusions

In conclusion, the study results highlight an existing problem related to the insufficient knowledge of surveyed medical students about brain death. It emphasises the significance of medical education in shaping the attitudes of future healthcare professionals towards brain death. Incorporating comprehensive and up-to-date teachings on brain death into the medical curriculum enable students to develop a deeper understanding and more informed perspectives on this complex subject, which may contribute to the challenge of insufficient organ donation.

### Supplementary Information

**Additional file 1.**

**Additional file 2**.

## Data Availability

The datasets used and analysed during the current study are available from the corresponding author on reasonable request.
