# Supplementary material for: The impact of clinical education on knowledge and attitudes towards brain death among Polish medical students – a cross-sectional study
Source: BMC Med Educ. 2023 Sep 14;23:669. doi: 10.1186/s12909-023-04637-y (PMC10503106; doi:10.1186/s12909-023-04637-y)
Supplement: Supplementary file 1 — Additional file 1. [file 12909_2023_4637_MOESM1_ESM.docx]

*Dear Students,*

*We invite you to complete the questionnaire on knowledge and worldview on the phenomenon of brain death. It consists of a theoretical part and a worldview part. Estimated time for completion is 15 minutes. Please do not use additional sources of knowledge.*

*The survey is aimed at medical students.*

*The survey is anonymous, voluntary, and the results will be analyzed collectively. At any point in the questionnaire, you can opt out of participating in its completion.*

*Team of the Department of Neurology at the Medical University of Wroclaw*

Indicate in the range provided the year of study (medical faculty) you are in:

- 1-3 years

- 4-6 years

**Knowledge test**

1 The current diagnostic criterion for brain death is:

● irreversible loss of brainstem function.

● irreversible loss of function of all brain cells

● irreversible loss of function of the cerebral cortex.

● irreversible loss of function of both hemispheres and the midbrain

● don't know

2 Which of the following symptoms excludes brain death?

● subtle periodic and rhythmic contractions of facial muscles.

● flexion movements of the fingers of the hand

● preservation of tendon reflexes.

● present pupillary reaction to light

● all of the above

3Is it possible to maintain pregnancy in women who have been diagnosed with brain death on artificial life support?

● No, because the brain stem has died.

● Yes, because the brain structures outside the brainstem are responsible for maintaining the pregnancy

● Don't know.

4 Which statement is true?

● Vegetative state, lock-in-syndrome and brain death are states in which loss of consciousness and awareness are observed.

● Vegetative state and locked-in syndrome are characterized by preserved awareness but lost consciousness, while people with confirmed brain death lack both consciousness and awareness.

● Lock-in syndrome is diagnosed when consciousness and awareness are preserved, vegetative state - awareness only, brain death - lack of both consciousness and awareness

● Don't know.

5 When does the death of the remaining tissues occur in a patient diagnosed with brain death?

● At the time of brainstem necrosis

● One day after brainstem necrosis.

● After cessation of artificial ventilation

● Don't know.

6 In whom can the suspicion of brainstem and brain death be raised and the procedure for its pronouncement initiated?

● A patient in whom irreversible cessation of circulation and respiration has occurred.

● A patient in a coma, artificially ventilated, with irreversible brain damage

● Patient, in a coma, artificially ventilated regardless of established brain damage

● Don't know

7 What conditions preclude the initiation of the procedure for declaring brain death?

● Poisoning by pharmacological agents, hypothermia, endocrine disorders, neonates <7 d. of age.

● Poisoning with pharmacological agents that have an inhibitory effect on the respiratory center in the brainstem, hypothermia

● Condition caused by prolonged hypoxia of brain tissue.

● No condition precludes the possibility of initiating the procedure for declaring brain death under existing law.

● Don't know

8 Is it always necessary to perform an instrumental examination in the procedure for pronouncing brain death?

● Yes

● No

● Don't know

9. Which of the reflexes is not checked during the pronouncement of brain death?

● pupillary response to light

● Babinski's

● pain response

● caloric test

● don't know

10. Who is on the committee for determining brain death (mark the most precise answer)

● 3 specialist physicians, including one in neurology or neurosurgery and one in anesthesiology and intensive care; in exceptional situations included in the law, the head of the department where the death occurred

● 3 specialist physicians, including one in neurology or neurosurgery and one in anesthesiology and intensive care

● 2 specialist physicians, one of whom is a specialist in anesthesiology and intensive care or neonatology, and the other in neurology, pediatric neurology or neurosurgery.

● any 2 physicians holding a License to Practice.

11 From whom can be taken organs for transplantation after death?

● From anyone who has not objected during his/her lifetime (with a reservation regarding minors)

● From whoever has given his consent during his lifetime

● From one who consented during his lifetime or whose relatives consented after his death

● Don't know

12 From which deceased, other than one who has been declared brain dead, can organs still be taken for transplantation?

● From the deceased who was diagnosed with irreversible cardiac arrest

● From the deceased in whom there are visible signs of death

● Organs can only be taken after brain death has been established, with artificial circulatory support

13 The current criteria for declaring brain death are mainly:

● Biological

● Ontological

● Formal-legal

● I don't know

**Worldview questionnaire**

1. Brain death is an irreversible condition.

2) If a patient is found to have the death of brain, life support should be terminated immediately (when transplantation is not planned).

3) If a pregnant woman is found to have symptoms of brain death, then her vital functions should be artificially maintained if the fetus has a chance of survival until the pregnancy is successfully terminated

4. The family should have the right to challenge the doctor's decision in brain death

5. Brain death is equivalent to cardiopulmonary death in the context of the death of an individual

6. All pronouncements of brainstem death should be preceded by an instrumental examination (EEG, cerebral flow, brainstem evoked potentials)

● strongly agree

● rather agree

● have no opinion

● rather disagree

● strongly disagree

Translated with www.DeepL.com/Translator (free version)

**Kwestionariusz światopoglądowy**

1. Śmierć mózgu jest stanem nieodwracalnym.
2. Jeśli stwierdzono u pacjenta śmierć mózgu należy niezwłocznie zakończyć podtrzymywanie życia (gdy nie jest planowana transplantacja).
3. Jeśli u kobiety w ciąży stwierdzono objawy śmierci mózgu to jej czynności życiowe powinny być sztucznie utrzymane, jeżeli płód ma szanse przeżycia do czasu pomyślnego rozwiązania ciąży
4. Rodzina powinna mieć prawo do zakwestionowania decyzji lekarza przy śmierci mózgu
5. Śmierć mózgu jest równoważna do śmierci krążeniowo-oddechowej w kontekście śmierci jednostki
6. Czy uważasz, że każde orzeknięcie śmierci pnia mózgu powinno być poprzedzone badaniem instrumentalnym (EEG, przepływ mózgowy, potencjały wywołane z pnia mózgu)?

● zdecydowanie zgadzam się

● raczej zgadzam się

● nie mam zdania

● raczej się nie zgadzam

● zdecydowanie się nie zgadzam
