# Supplementary material for: The impact of clinical education on knowledge and attitudes towards brain death among Polish medical students – a cross-sectional study
Source: BMC Med Educ. 2023 Sep 14;23:669. doi: 10.1186/s12909-023-04637-y (PMC10503106; doi:10.1186/s12909-023-04637-y)
Supplement: Supplementary file 2 — Additional file 2. [file 12909_2023_4637_MOESM2_ESM.docx]

**Supplementary Table. Scheme of conduct of the survey**

Asking the question about medical students' knowledge, beliefs and attitudes and whether it is changing during their studies?

Developing the online quantitative computer-assisted web interview (CAWI) survey

Spreading the information about the anonymous survey among students of four medical universities in Poland (Wroclaw, Poznan, Katowice, and Warsaw). Participants were provided with a unique link to access the questionnaire.

Collection of data. Validation procedures were implemented to ensure the accuracy and reliability of the collected data.

Analysis of data. Descriptive statistics, correlation analyses.
